# The Importance of MTHFR C677T/A1298C Combined Polymorphism in Deep Vein Thrombosis: A Case Report

**DOI:** 10.7759/cureus.29265

**Published:** 2022-09-17

**Authors:** Marcia Machado, Daniela Neto, Silvia Nunes, Cristina Cunha, Carlos Fernandes, Glória Alves, Jorge Cotter

**Affiliations:** 1 Internal Medicine, Hospital da Senhora da Oliveira Guimarães, Guimarães, PRT

**Keywords:** oral contraceptive pills side effect, porto-splenic-mesenteric thrombosis, mesenteric venous thrombosis, abdominal pain, increase in homocysteine, mthfr a1298c mutation, mthfr c677t, testing for thrombophilia, mthfr mutation, acute portal vein thrombosis

## Abstract

A 37-year-old woman presented in the emergency room with abdominal pain and nausea for about three weeks. She had no known risk factors for venous thromboembolism beyond taking oral contraceptives as a regular medication. Computed tomography (CT) scan revealed portal, superior mesenteric and splenic vein thrombosis. Thrombophilia tests were negative, except for the presence of heterozygosity for mutation of the methylenetetrahydrofolate reductase (*MTHFR*) gene. Homocysteine levels and folic acid were normal. Anticoagulation was started. Follow-up CT after eight months showed cavernous transformation of the portal vein.

## Introduction

Portal vein thrombosis (PVT) occurs when there is an occlusion of the portal vein with or without extension of splenic or superior mesenteric veins. It is responsible for about 5-10% of cases of portal hypertension. Symptoms often depend on the location and size of the clot. Vein thrombosis can result from malignancy, prothrombotic states such as thrombophilia, pregnancy, hormone replacement/oral contraceptives, myeloproliferative disorders, and autoimmune diseases [1]. Mutation in the methylenetetrahydrofolate reductase (*MTHFR*) gene is the most frequent cause of moderate increase in homocysteine [2]. Homocysteine causes vascular endothelial dysfunction, activates the clotting system, and inhibits the fibrinolytic system [3]. We describe the case of a 37-year-old woman with abdominal pain associated with PVT and an *MTHFR* C677T/A1298C combined polymorphism with normal homocysteine levels.

## Case presentation

**Figure 1:**
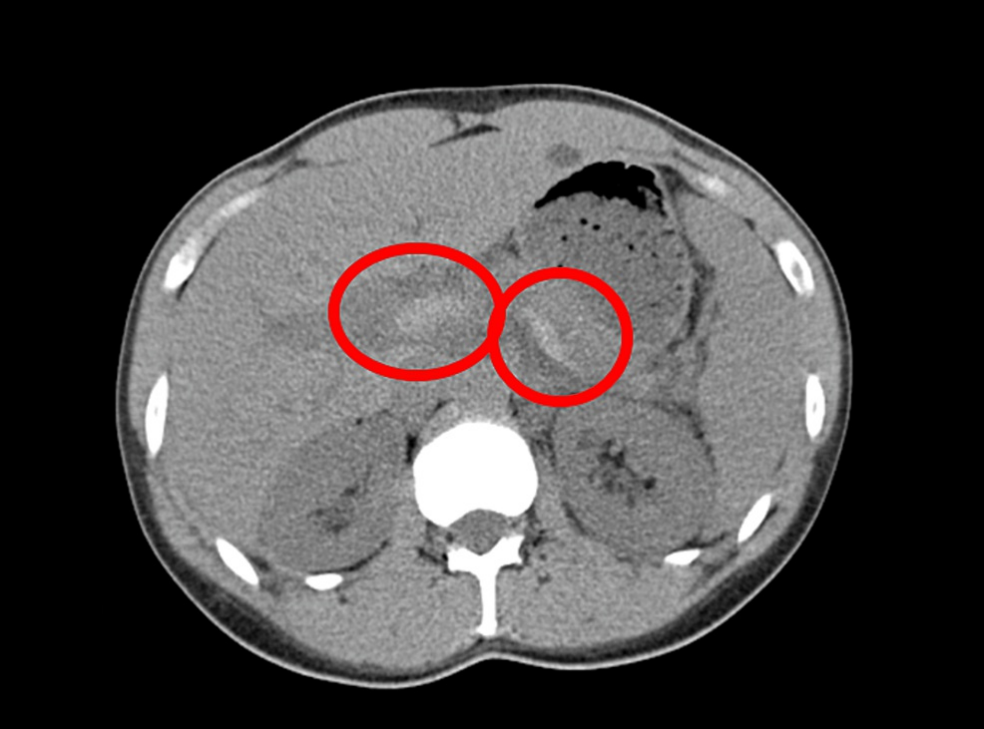
CT scan of the abdomen showing splenic and portal vein thrombosis (circle) CT: computed tomography

A 37-year-old caucasian woman presented in the ER with nausea and abdominal pain. The pain had started three weeks earlier as epigastric pain and evolved to diffuse abdominal pain with increasing intensity to 7/10, with no relief after analgesics. She had no other symptoms. She had no comorbidities. Her usual medication was combined estrogen-progestin oral contraceptives for 20 years. She had an active lifestyle. She did not consume alcohol, tobacco or any other type of drug of abuse. She had no family history of thromboembolic events. The patient appeared to be in distress due to abdominal pain. Her blood pressure was 122/74 mmHg, pulse rate 95 bpm, respiratory rate of 26 cpm, and temperature of 36,1ºC. Abdominal palpation was mildly painful in the upper quadrants, with no other remarkable signs on physical examination. Blood tests had no remarkable changes. Abdominal ultrasound showed mesenteric, splenic, and upper portal thrombosis and CT angiography confirmed the thrombotic event and excluded other changes (Figure 1). 

She was admitted and was anticoagulated with enoxaparin 1 mg/kg every 12 hours. An endoscopy and colonoscopy, chest CT scan, mammography, ultrasonography of the breast, neck, and thyroid, and myelogram were done with no suspicious results. Tests for serological markers for viral hepatitis B, hepatitis C, and HIV were negative. A genetic test showed normal factor V Leiden and prothrombin genotypes, and mutation in heterozygosity for the *MTHFR* C677T/A1298C. Antithrombin, protein C and S activity were normal; lupus anticoagulant was not detected and anticardiolipin antibodies and anti-glycoprotein B antibodies were negative; The homocysteine level was 7 μmol/L (reference range 4-12) and folic acid was normal. She was discharged with a prescription for warfarin. Eight months after discharge, she was asymptomatic and CT scan showed cavernous transformation of the portal vein.

## Discussion

The portal vein results from the confluence of the splenic and superior mesenteric veins and drains directly into the liver [4]. PVT is observed in 0,6-16% of patients with liver cirrhosis and it is responsible for 5-10% of all cases of portal hypertension. Acute PVT usually causes abdominal pain and the physical findings are not remarkable, except in case of intra-abdominal inflammation, intestinal infarction, or perforation. Venous thromboembolism is a disease of multifactorial etiology, resulting from the interaction of genetic factors and environmental factors [1]. The use of oral contraceptive pills may promote thrombosis by increasing levels of coagulation factors and decreasing levels of coagulation inhibitors [5]. Furthermore, combined oral contraceptives, containing ethinylestradiol and a progestogen, have more impact in women with inherited thrombophilia (antithrombin, protein C and protein S deficiencies, factor V Leiden, and prothrombin G20210A mutation) [6]. *MTHFR* variants are also associated with an increased risk of venous thromboembolism. MTHFR is a crucial enzyme in the remethylation pathway of homocysteine metabolism and molecular defects can result in enzyme deficiency and, consequently, in hyperhomocysteinemia, which is associated with an increased risk of venous thromboembolism [2]. Homocysteine causes vascular endothelial dysfunction, activates the clotting system, and inhibits the fibrinolytic system [3]. On the other hand, there is evidence that the presence of a combined mutation in heterozygosity for the C677T and A1298C of the *MTHFR* gene is associated with venous thromboembolism even without associated hyperhomocysteinemia [7]. Anticoagulation is the mainstay of therapy for PVT, unless it is contraindicated, to enable portal vein recanalization and prevent of portal hypertension [8]. Portal hypertension is a complication of PVT and its consequences include ascites and esophageal varices. Recanalization of the portal vein is expected to occur up to six months and of the mesenteric and splenic veins up to 12 months of follow-up. So anticoagulation therapy should be given for at least six months and a CT scan should be performed to assess portal venous system recanalization at 6-12 months of follow-up. If effective portal vein recanalization does not occur, the collateral veins dilate and become serpiginous (cavernous transformation of the portal vein) [9]. 

## Conclusions

This case report suggests that carriers of a combined mutation in heterozygosity for the C677T and A1298C of the *MTHFR* gene who use oral contraceptives have an increased risk of venous thromboembolism. Further studies are needed to assess the relationship between inherited prothrombotic conditions, including *MTFR* genotype, and the use of oral contraceptives that can potentiate their prothrombotic effect. Genetic testing may be important to assess thrombotic risk before starting oral contraceptives. 
